# Case Report: Postsurgical hallux varus in which metatarsophalangeal joint arthrodesis was useful

**DOI:** 10.12688/f1000research.131495.1

**Published:** 2023-03-28

**Authors:** Naoki Kondo, Tetsuya Igarashi, Tomoya Inukai, Hiroyuki Kawashima

**Affiliations:** 1 Division of Orthopedic Surgery, Department of Regenerative and Transplant Medicine, Niigata University Graduate School of Medical and Dental Sciences, Niigata, Niigata, 951-8510, Japan

**Keywords:** Hallux valgus; Metatarsophalangeal joint arthrodesis; Iatrogenic hallux varus; Cup and cone reamer; Shortening oblique osteotomy

## Abstract

A 74-year-old Japanese woman who underwent Mann’s procedure with fibular sesamoidectomy for left hallux valgus 21 years ago complained of left hallucis pain. She was diagnosed with iatrogenic hallux varus and hammer toe deformities. Metatarsophalangeal joint arthrodesis and shortening oblique osteotomy were performed. After surgery, the hallux valgus angle improved from -28° to 0°, and the intermetatarsal angle between the first and the second metatarsus improved from 0° to 6°. The Japanese Society for Surgery of the Foot score improved from 75 to 81 points. She could walk without pain and sustained no deformity at 4 years after the surgery.

## Introduction

Hallux varus is a rare foot deformity due to iatrogenic, post-traumatic, idiopathic, inflammatory, spontaneous, or congenital pathologies. In particular, the iatrogenic type is the most common cause of hallux varus. ^1^^,^^2^ Multiple studies reported that postsurgical hallux varus was observed in 2%–15.4% of cases. ^3^

Post-surgically observed hallux varus is attributed to overcorrection of the hallux valgus deformity. This includes excessive removal of the medial osteophyte and over-release of adductor halluces tendons, transmetatarsal ligament, and lateral metatarsophalangeal (MTP) joint capsule. ^1^ The incidence of iatrogenic hallux varus after surgery of hallux valgus is very low, and there is a paucity of reports associated with the treatment strategy.

Herein, we report a novel case of postsurgical hallux varus deformity. We performed revision surgery, i.e., MTP joint arthrodesis for hallucis and shortening oblique osteotomy for the lesser toes. Four years after surgery, the patient was satisfied, functionally good, and experienced no pain upon standing or walking. No postoperative callosity was detected.

## Case report

**Figure 1.**
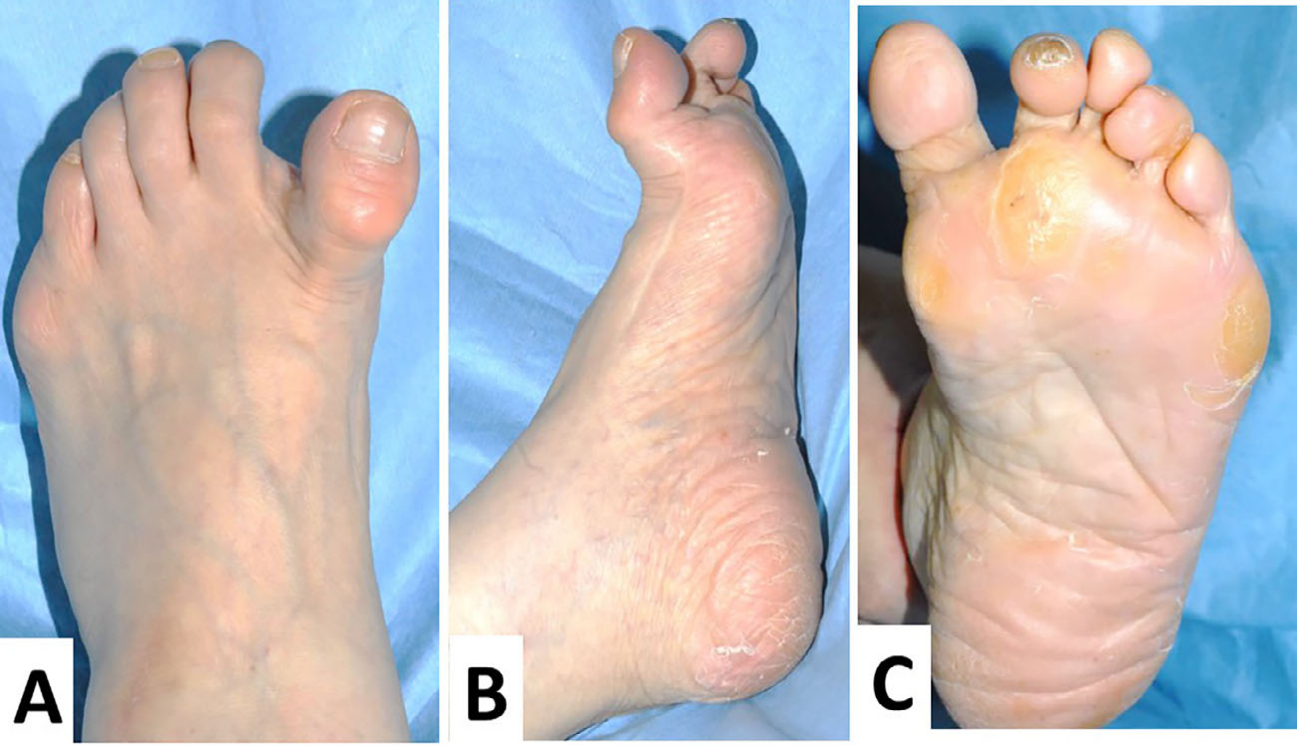
Preoperative macroscopic findings of the left foot. The extensor hallux longus is very tense, with the metatarsophalangeal (MTP) joint hyperextended and the interphalangeal (IP) joint in a flexed position, forming a so-called “cock-up deformity” (A). In the lateral view, a preoperative operation scar is clearly detected (B). In the plantar view, the varus deformity is clearly observed, and no callosity is detected (C).

**Figure 2.**
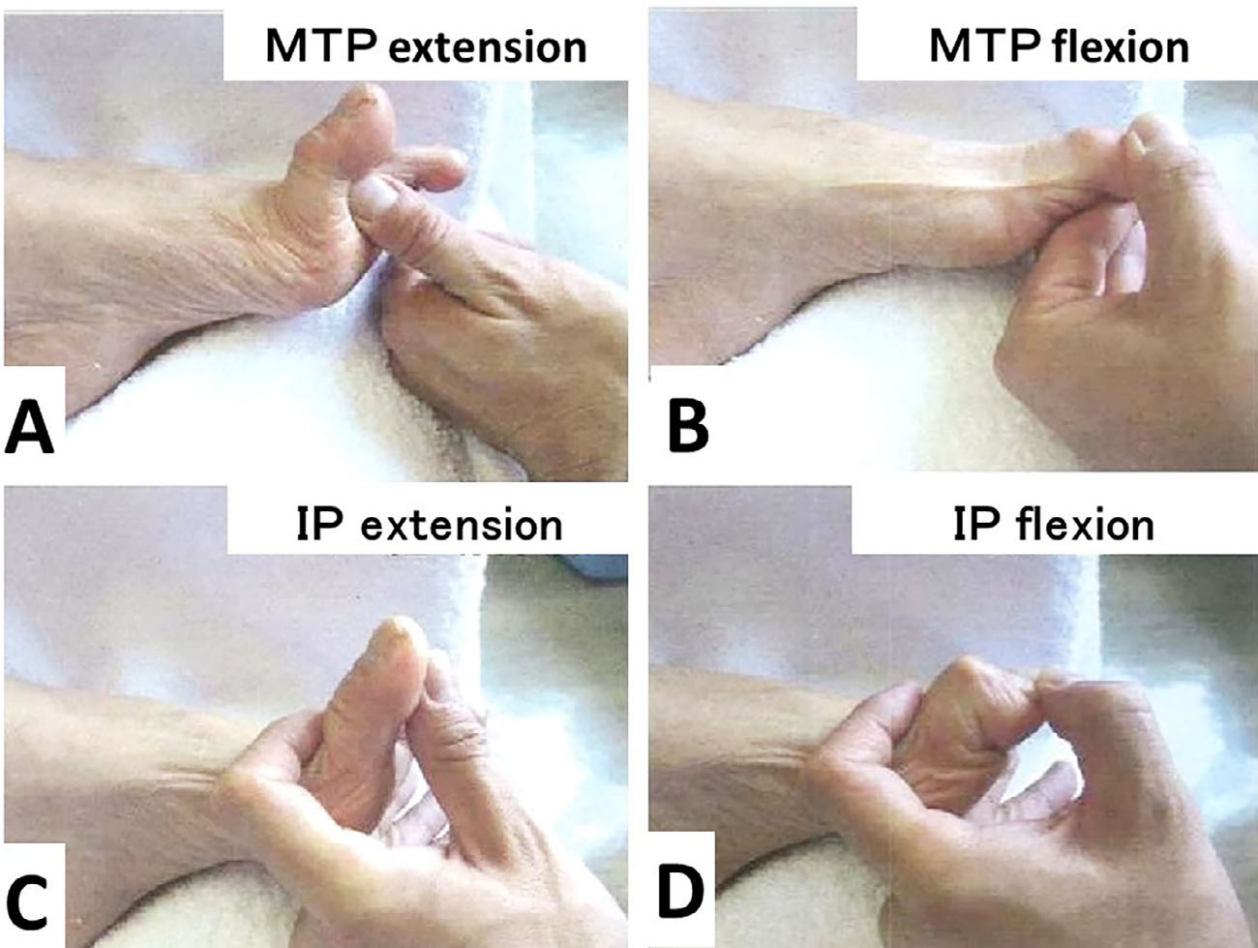
Passive motion of metatarsophalangeal (MTP) and interphalangeal (IP) joints of the left hallucis. The passive extension and flexion of the MTP joint are 90° (A) and -10° (B), respectively. The passive extension and flexion of the IP joint are -20° (C) and 90° (D), respectively.

A 74-year-old Japanese woman visited our clinic with complaints of left hallucis pain and concerns about medial deviation. Twenty-one years prior, the patient underwent Mann’s procedure with bunionectomy and with fibular sesamoidectomy, a surgical operation for bilateral hallux valgus. After the operation, the MTP joint surface deviated medially in a hyperextended position ( Figure 1A– C). In addition, the second and fourth toes demonstrated hammer toe deformities ( Figure 1A and C). First, an orthosis was applied; however, medial deviation and pain in her MTP joint worsened six months after the orthosis application. Hence, surgical intervention was decided on. Upon physical examination, the left MTP joint of the patient was swollen, tender, and erythematous. Extensor hallucis longus was very tense, the MTP joint was hyperextended, and the interphalangeal (IP) joint was in a flexed position, resulting in the “cock-up deformity” of hallux varus ( Figure 2A– D). Lateral instability of the MTP joint of the hallux was not detected.

The Japanese Society for Surgery of the Foot (JSSF) hallucis score ^4^ is often used in Japan as a means to evaluate the function of the ankle and foot. The patient’s score was 75 out of 100 points (pain, 30 out of 30; deformity, 17 out of 25; range of motion, 15 out of 15; gait, 10 out of 20; and activity of daily life, 3 out of 10).

Regarding the range of motion, the MTP joint was very stiff and showed extension contracture (-90° in extension and -30° in flexion), but the IP joint remained flexible (-5° in extension and 90° in flexion) ( Figure 2A– D).

**Figure 3.**
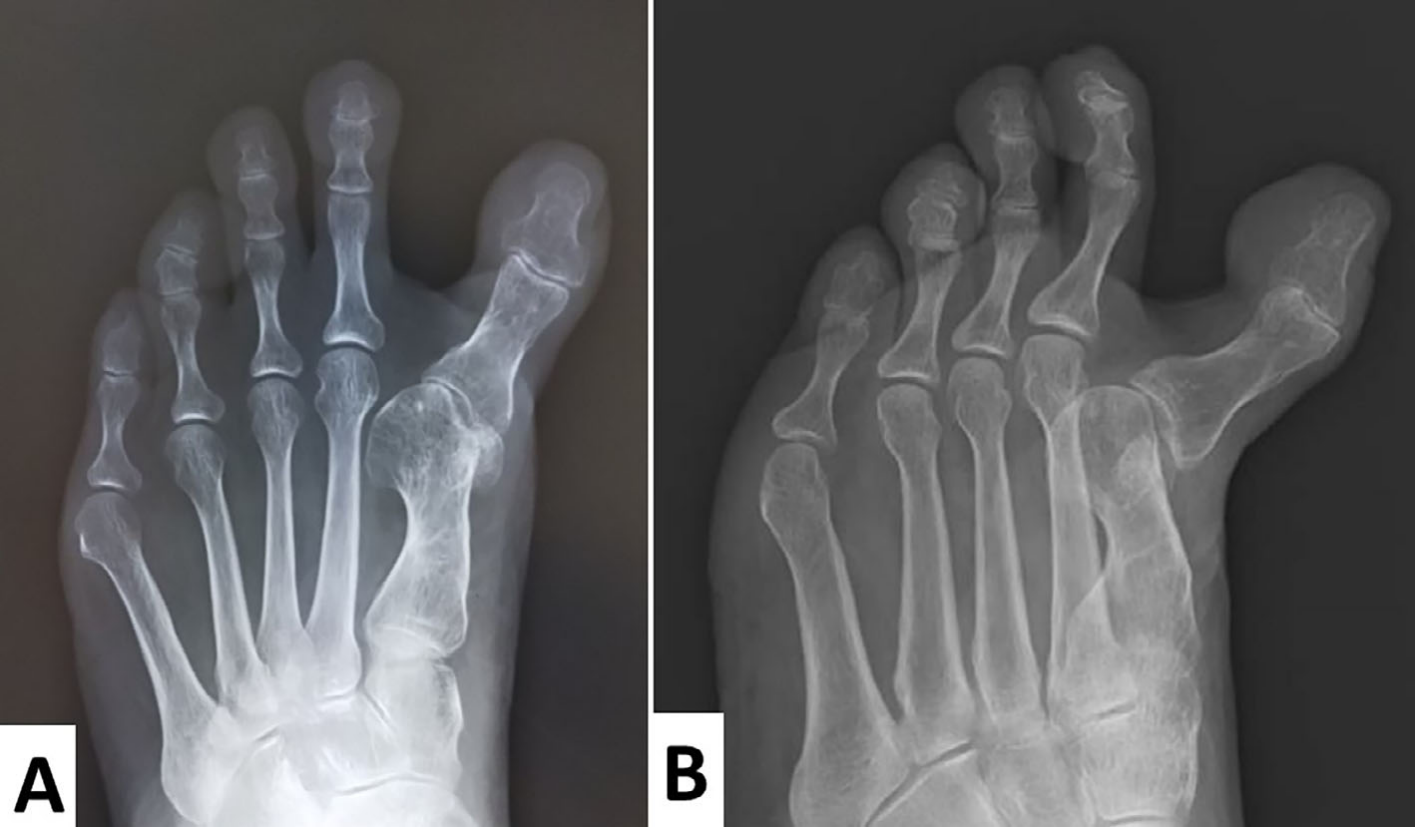
Passive motion of metatarsophalangeal (MTP) and interphalangeal (IP) joints of the left hallucis. Radiographs show that the hallux valgus angle was -28°. The intermetatarsal angle, which should be approximately 6°–9°, is 0°. This indicates that the first and second metatarsal bones are parallel. The tibial sesamoid has shifted medially and the fibular sesamoid is absent. Excessive medial eminence resection might have been performed.

Radiographs showed that the hallux valgus angle (HVA) was -28° ( Figure 3A). The intermetatarsal angle between the first and the second metatarsus (M1M2A) was 0° (normal range, 6°–9°), which meant that the first and second metatarsal bones were parallel ( Figure 3A). As the tibial sesamoid shifted medially, and the fibular sesamoid was absent, excessive medial eminence resection might have been performed. An oblique view of the foot demonstrated that the proximal phalanx subluxated dorsally ( Figure 3B).

**Figure 4.**
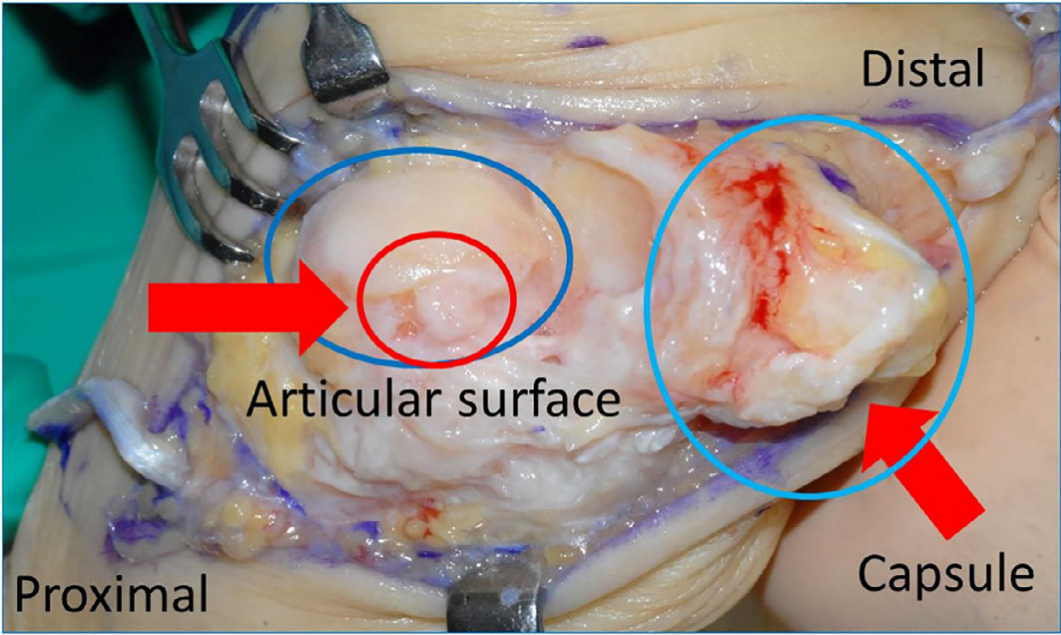
Macroscopic operative findings. The medial portion of the articular surface reveals severe degenerative change. Degenerative change is also seen in the capsule.

In this case, MTP joint arthrodesis, medial capsular release, and EHL tendon lengthening were performed. For the second and fifth toes, a shortening oblique osteotomy was performed. The intraoperative macroscopic findings revealed that the medial portion or the articular surface was impacted by the severe degenerative change. The degenerative changes were also observed in the capsule ( Figure 4).

A cup and cone type reamer (Wright Medical, Tokyo, Japan) was used to preserve the length of the hallux as much as possible. The metatarsal articular surface was reamed to a cup-shaped surface, and the proximal phalanx articular surface was recreated with a cone-shape.

**Figure 5.**
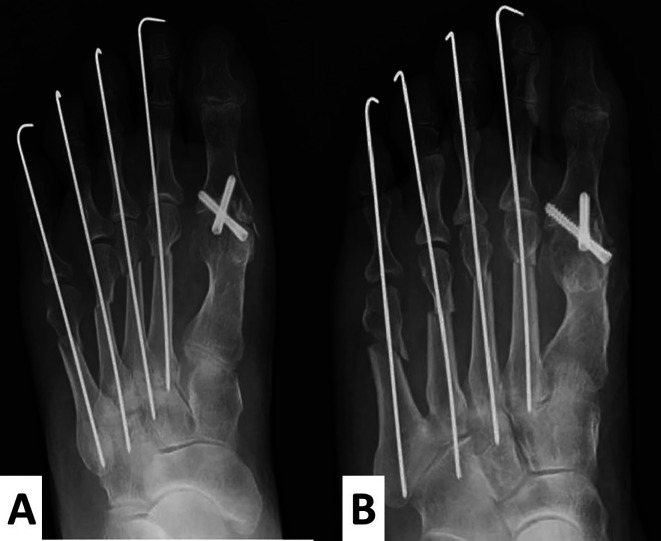
Radiographs after the operation. A cup and cone reamer are used to preserve the length of the hallux as much as possible. The hallux valgus angle is fixed at 0°, and the 1 ^st^ proximal phalanx is dorsally fixed at 15° to the metatarsal bone. M1M2A shows 6°. For the lesser toes, a shortening oblique osteotomy is performed (A, anteroposterior view; B, oblique view).

The hallux valgus angle was fixed at 0°, and the first proximal phalanx axis was dorsally fixed at 15° to the metatarsal bone axis. Two full-thread Acutrak ^®^ screws (Nihon Medical Next Co. Ltd, Tokyo, Japan) were inserted at the fixed position in a crisscross fashion. For the lesser toes, a shortening oblique osteotomy was performed ( Figure 5A and B). The postoperative radiographs showed that M1M2A was 6° and HVA was 0° ( Figure 5A).

Temporal fixation of the lesser toes with Kirchner wires (1.2 mm in diameter) was performed, and then the wires were removed after three weeks. Arch support was applied, and full weight gait exercise was performed.

**Figure 6.**
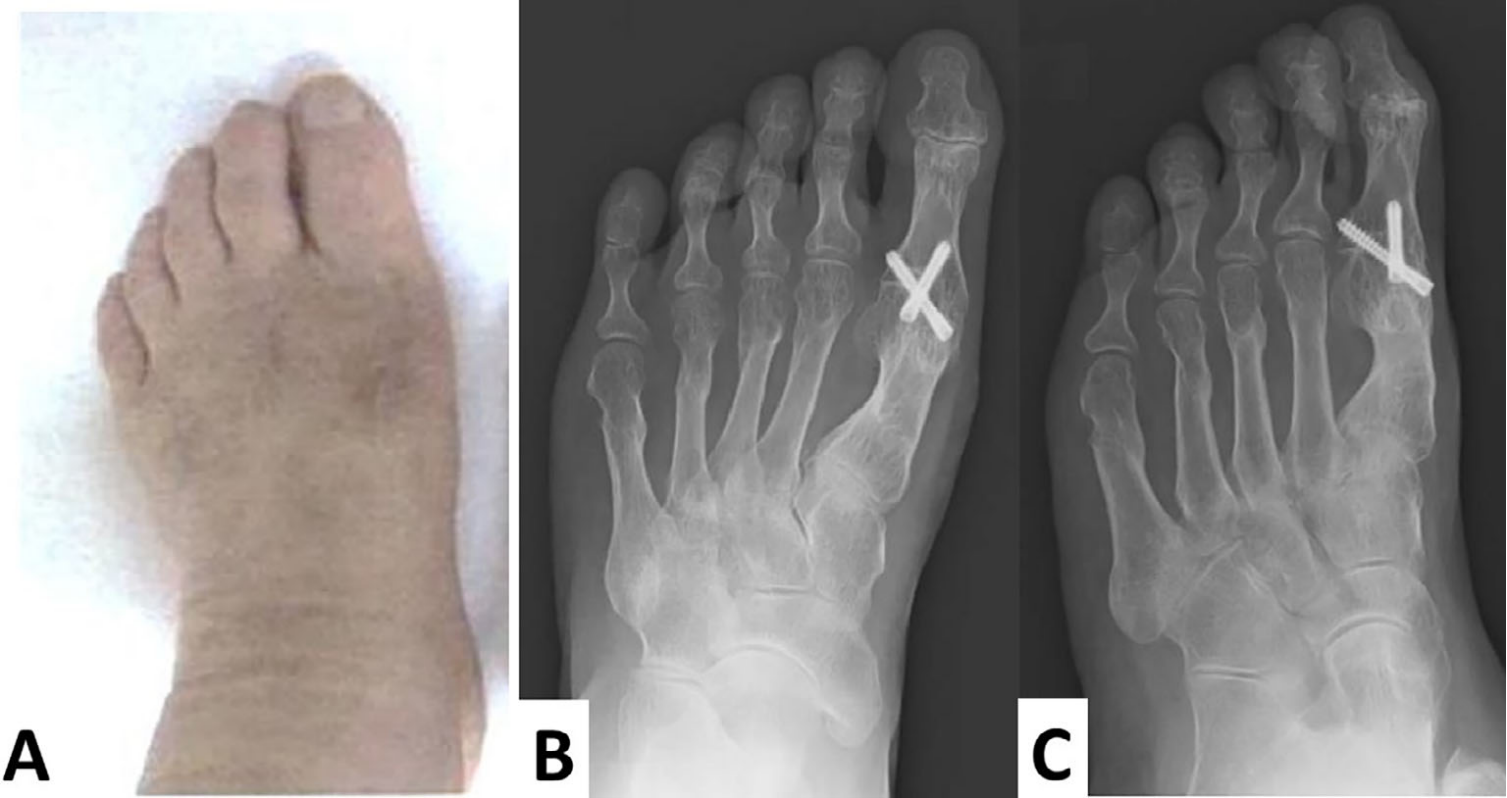
Macroscopic findings and radiographs are taken four years after the operation. No recurrence of hallux varus and hallux valgus is observed (A). The screws are intact, in place, and no valgus or varus deformities are apparent. Additionally, each osteotomized lesser toe is united (B, anteroposterior view; C, oblique view).

The screws remained intact and in place, and no valgus or varus deformities were apparent four years after surgery ( Figure 6A– C).

Post-operative evaluation of the JSSF score of hallucis was 81 out of 100 (pain, 30 out of 30; deformity, 23 out of 30; range of motion, 5 out of 15; gait, 20 out of 20; and activity of daily life, 3 out of 10), which showed a six-point increase. A six-point increase in deformity and a 10-point increase in walking abilities were noted; however, a 10-point decrease in range of motion was observed. No deformities were apparent and pain that worsened during movement was relieved.

## Discussion

Hallux varus is a clinical condition characterized by the medial deviation of the great toe at the MTP joint. Iatrogenic hallux varus is caused by an imbalance between the various bone, tendon, and capsule-ligament structures of the first MTP joint, including a progressive medial deviation of the hallux. ^5^

The causes of iatrogenic hallux varus are 1) overstitching of the medial joint capsule, 2) medial deviation of the tibial sesamoid, 3) over-traction by the abductor hallucis muscle due to lateral ligament complex release, 4) postoperative dressing in varus position of the hallux metatarsophalangeal joint, and 5) over-excision of the medial bony protrusion of hallux metatarsus. The patient in our case exhibited the second and fifth causes. ^6^

Akhtah *et al.* reported that the hallux varus was mainly classified into three types: osseous, myoligamentous, and combined. ^7^ In our case, the hallux varus was the combined type.

The surgical procedures for iatrogenic hallux varus depend on salvaging the MTP joint of the hallucis. When the MTP joint can be preserved, the medial joint capsule is released, and the MTP joint is reconstructed using procedures such as reverse chevron osteotomy and tendon transfer of abductor hallucis. ^6^

Leemrijse *et al.* recently reported a treatment algorithm. ^5^ According to this algorithm, in the case of a stiff MTP and mobile IP joint of the hallux, MTP joint arthrodesis is indicated. The present case also demonstrated both stiff MTP and mobile IP. Subsequently, MTP joint arthrodesis was performed.

When MTP cannot be preserved, MTP joint arthrodesis is indicated; however, MTP joint mobility is sacrificed. In this case, severe joint incongruity and irreversible flexion contracture of the MTP joint were detected. In addition, intraoperative findings revealed that the MTP joint surface degenerated at both the metatarsal and proximal phalanx articular surfaces ( Figure 4). Therefore, MTP joint arthrodesis was selected.

Leemrijse *et al.* and Piat *et al.* described that MTP fusion is the most reliable solution and is inevitable if the joint is stiff or degenerative. ^5^^,^^8^

MTP joint arthrodesis for hallux varus also significantly improved both the average 1–2 intermetatarsal angle from 4.8° to 8.4° and HVA from -20.7° to 8.1° in 26 patients (29 feet). ^9^ In our case, M1M2 A improved from 0° to 6° and HVA improved from -28° to 0° postoperatively ( Figures 3A and 5A).

Tourne *et al.* reported 14 cases of hallux varus. Each case showed medial arthrolysis of the MTP joint. Of 14 patients, five were treated with a reconstruction procedure of the lateral ligament accompanied by the medial release. Thereafter, nine patients were treated with MTP joint arthrodesis in case the MTP joint was stiff and arthrosis was present. According to the 100-point scoring system, the results were excellent in 56% and good in 44% of the patients with MTP joint arthrodesis. ^10^

The guidelines for our cases were as follows: 1) V-shaped incision was used for the dorsal MTP joint capsule. Thereafter, the articular surface was sufficiently exposed and medial tightness was thoroughly released. Fibular sesamoid was also released and relocated; subsequently, the V-shaped flap was tightly repaired after the MTP joint fusion. 2) The MTP joint level of hallucis after the primary surgery was much shorter than those of the lesser toes. To correct the imbalance of the MTP joint line between hallucis and lesser toes, and to prevent postsurgical metatarsalgia, we used a cup and cone reamer to minimize bone excision of the hallux metatarsus. Ball and cup reamer and osteosynthesis with pure titanium staples have been reported to yield good results in 54 patients with hallux valgus. ^11^ In addition, we performed shortening oblique osteotomy for lesser toes. 3) EHL elongation was performed because the EHL tendon became shortened due to flexion contracture of hallucis.

In conclusion, MTP joint arthrodesis using a cup and cone reamer minimized the shortening length of the metatarsal bone and proximal phalanx bone of the hallux. Additionally, it enabled stabilization in walking and bearing on the foot, resulting in good functional outcomes for this iatrogenic hallux varus case.

## Consent

Written informed consent for publication of their clinical details and clinical images was obtained from the patient.

## Data Availability

No data are associated with this article.
