# Supplementary material for: Case Report: Postsurgical hallux varus in which metatarsophalangeal joint arthrodesis was useful
Source: F1000Res. 2024 Jul 9;12:344. Originally published 2023 Mar 28. [Version 2] doi: 10.12688/f1000research.131495.2 (PMC11979576; doi:10.12688/f1000research.131495.2)
Supplement: Peer review history

# Reviewer report: Reviewer response for version 2

**DOI:** 10.5256/f1000research.168017.r372510

**Published:** 2025-04-08

**Authors:** Kenichiro Nakajima

**Affiliations:** 1 Center for Foot and Ankle Surgery, Department of Orthopedic Surgery, Yashio Central General Hospital, Saitama, Japan

This case report describes a patient who developed iatrogenic hallux varus following hallux valgus surgery and subsequently underwent arthrodesis of the first metatarsophalangeal joint, achieving good outcomes. Overall, this paper is well-written; however, some terminology concerns should be addressed.

 Abstract

 - Line 4: Please change “joint arthrodesis and shortening oblique osteotomy” to “joint arthrodesis for the hallux varus and shortening oblique osteotomies for the hammer toes”.

 Introduction

 - Paragraph 2, line 2: Please change “osteophyte” to “eminence”.

 - Paragraph 2, line 3: Please change “tendons” to “tendon”.

 - Paragraph 3, line 2: Please change “hallucis” to “the hallux”.

 - Paragraph 3, line 2: Please change “osteotomy” to “osteotomies”.

 Case report

 - Paragraph 1, line 9: Please change “Extensor hallucis longus” to “The extensor hallucis longus (EHL)”.

 - Figure 2 legend: Please change “the left hallucis” to ”the left hallux”.

 - Paragraph 4, line 4: Please change “the first and the second metatarsus” to “the first and second metatarsals”.

 - Figure 3 legend: Please change “the left hallucis” to “the left hallux”.

 - Paragraph 7 (Beginning with “The hallux valgus”), line 4: Please change “a shortening oblique osteotomy was” to “shortening oblique osteotomies were”.

 - Paragraph 8 (Beginning with “Dorsal curved”), line 3: Please change “osteotomy” to “osteotomies”.

 - Paragraph 8, line 6: Please change “osteophyte in the metatarsal head was” to “protruding portions of the metatarsal heads were”.

 - Paragraph 10, line 2: Please change “arch support” to “an arch support”.

 - Paragraph 12 (The last paragraph), line 1 : Please change “hallucis” to “the hallux”.

 - Paragraph 12, line 5: Please change “that worsened” to “that had worsened”.

 Discussion

 - Paragraph 2, line 5: Please change “The patient in our case exhibited the second and fifth causes.” to “The patient in this case appeared to exhibit hallux varus due to the second and fifth causes”.

 - Paragraph 10 (Beginning with “The guidelines”), line 1: Please change “our cases” to “our case”.

 - Paragraph 10, line 5: Please change “hallucis” to “the hallux”.

 - Paragraph 10, line 6: Please change “hallucis and lessor toes” to “the hallux and the lessor toes”.

 - Paragraph 10, line 8: Please change “the hallux metatarsus” to “the first metatarsal”.

 - Paragraph 10, line 10: Please change “oblique osteotomy for lessor toes” to “oblique osteotomies for the lessor toes”.

 - Paragraph 10, line 11: Please change “hallucis” to “the hallux”.

Are enough details provided of any physical examination and diagnostic tests, treatment given and outcomes?

Yes

Is the case presented with sufficient detail to be useful for other practitioners?

Yes

Is sufficient discussion included of the importance of the findings and their relevance to future understanding of disease processes, diagnosis or treatment?

Yes

Is the background of the case’s history and progression described in sufficient detail?

Yes

Reviewer Expertise:

foot and ankle surgery

I confirm that I have read this submission and believe that I have an appropriate level of expertise to confirm that it is of an acceptable scientific standard.

# Reviewer report: Reviewer response for version 2

**DOI:** 10.5256/f1000research.168017.r300623

**Published:** 2024-07-26

**Authors:** Inderjeet Singh Rikhraj

**Affiliations:** 1 Singapore General Hospital, Outram Rd, Singapore

Introduction

 A medial osteophyte is not removed, it is a bunionectomy. - This is not corrected.  Others all ok.

 Please get authors to change this and can be indexed.

Are enough details provided of any physical examination and diagnostic tests, treatment given and outcomes?

Yes

Is the case presented with sufficient detail to be useful for other practitioners?

Yes

Is sufficient discussion included of the importance of the findings and their relevance to future understanding of disease processes, diagnosis or treatment?

Yes

Is the background of the case’s history and progression described in sufficient detail?

Yes

Reviewer Expertise:

Outcomes research

I confirm that I have read this submission and believe that I have an appropriate level of expertise to confirm that it is of an acceptable scientific standard.

# Response

**Published:** 2024-07-27

**Authors:** Naoki Kondo

**Affiliations:** Niigata University, Japan

Dear, reviewer 2, 

 Thank you for your comment.

 I would like to revise Introduction.

 I would like to correct  "medial bunionectomy" from "excessive removal of the medial osteophyte" in the revised manuscript. 

 Best regards,

 July 27th, 2024

 Naoki Kondo

# Reviewer report: Reviewer response for version 1

**DOI:** 10.5256/f1000research.144343.r192634

**Published:** 2024-06-14

**Authors:** Inderjeet Singh Rikhraj

**Affiliations:** 1 Singapore General Hospital, Outram Rd, Singapore

Well written report on a rare complication. Highlights the problem of an iatrogenic hallux varus due to resection of the fibula sesamoid. The JSSF score have improved. It would be good to highlight the Minimal Clinical Important Difference (MCID) of the JSSF to point out the significance of the improvement.

 In the abstract, it is stated that the fibular sesamoidectomy was done 21 years ago. However, in the discussion, paragraph 10, line 2, it is stated that the fibular sesamoid was released and relocated. How is it possible to do this when the fibular sesamoid was resected?

Introduction

 A medial osteophyte is not removed, it is a bunionectomy.

Case Report

 "First, an orthosis was applied; however, medial deviation and pain in her MTP joint worsened six months after the orthosis application" Please rewrite and put a full top and not a colon.

 "As the tibial sesamoid shifted medially, and the fibular sesamoid was absent, excessive medial eminence resection might have been performed" This could also result from erosion as the proximal phalanx is abutting the medial aspect of the first metatarsal head, as the index surgery was done many years ago

Discussion

 "The surgical procedures for iatrogenic hallux varus depend on salvaging the MTP joint of the hallucis." this sentence is redundant.

 Para 2 " The patient in our case exhibited the second and fifth causes" what does this line mean?

 Combine paras 4 & 5 and rephrase as there is repetition and rewrite it

 Para 10 should be removed and put under Case Report. It is only 1 case report, it cannot be plural.

 In Abstract and Introduction, you state that a fibular sesamoidectomy was performed in the initial surgery. How can your surgical procedure state "Fibular sesamoid was also released and relocated"

 Last para "Additionally, it enabled stabilization in walking and bearing on the foot" There should be the words "weight -bearing" and not " bearing" This sentence should be rewritten to state that the foot was stable in weightbearing and walking

 The surgery was done well especially of the lesser toes.

Are enough details provided of any physical examination and diagnostic tests, treatment given and outcomes?

Yes

Is the case presented with sufficient detail to be useful for other practitioners?

Yes

Is sufficient discussion included of the importance of the findings and their relevance to future understanding of disease processes, diagnosis or treatment?

Yes

Is the background of the case’s history and progression described in sufficient detail?

Yes

Reviewer Expertise:

Outcomes research

I confirm that I have read this submission and believe that I have an appropriate level of expertise to confirm that it is of an acceptable scientific standard, however I have significant reservations, as outlined above.

# Response

**Published:** 2024-06-16

**Authors:** Naoki Kondo

**Affiliations:** Niigata University, Japan

I appreciate your precious comment.　

 I reconfirmed the operative record. As you pointed out, fibular sesamoidectomy had been performed.

 I corrected from fibular sesamoid to tibial sesamoid In Discussion part. The released sesamoid was tibial one.

 Including the reviewer comment 1, I totally revised the manuscript and I showed the corrected parts were colored with red.

# Reviewer report: Reviewer response for version 1

**DOI:** 10.5256/f1000research.144343.r236596

**Published:** 2024-02-08

**Authors:** Jun-Ichi Fukushi

**Affiliations:** 1 National Hospital Organization Kyushu Medical Center, Fukuoka, Japan

The report describes a case with postsurgical hallux varus, which can be a challenging issue for foot and ankle surgeons. This case report is overall well written, and the reviewer understands the usefulness of MTP joint arthrodesis as a reliable solution for iatrogenic hallux varus.  

**Points of reviews.**

**Introduction**

 1.In the first paragraph, the authors documented the incidence of postsurgical hallux varus as 2%-15.4%, which does not seem like “very low” to the reviewer. Therefore, the authors are required to reconsider the expression regarding the incidence of iatrogenic hallux varus in the second paragraph.

**Case report**

 1.JSSF hallux scale consists of three major items, which are pain, function, and alignment. The JSSF scale used in the present study consists of five items, including pain, deformity, ROM, gait, and ADL, which are used for JSSF RA foot and ankle scale (Ref 1). The authors are required to explain the reason why they used JSSF RA scale instead of hallux scale.

 2.How did the authors evaluate ROM of the hallux? Although the authors mentioned as “the MTP joint was very stiff” and “extension contracture”, the item for ROM was 15 (out of 15 points) preoperatively. The flexion of the hallux was mentioned as -10 in the legend of figure 2, and -30 in the main text.

 3.In the 7 ^th^ paragraph, it is also desirable to address the documentation regarding lesser toe shortening osteotomy in more detail.

**Discussion**

 1.The third paragraph (starting with “Akhtah et al”..) consisting of two sentences may be unnecessary.

 2.In the following four paragraphs (4 ^th^ to 7 ^th^), salvage procedures for iatrogenic hallux varus were discussed. However, some of the description referring Leemrijse et al was redundant. Adding the documentation of the indication for MTP preservation as well as MTP arthrodesis will provide readers with a better understanding of treatment algorithm advocated by Leemrijse.

 3.Was there fibular sesamoid remained? In the paragraph 10, the authors mentioned as “Fibular sesamoid was also released..” On the other hand, fibular sesamoidectomy was supposed to be performed 21 years prior to the revision surgery.

Are enough details provided of any physical examination and diagnostic tests, treatment given and outcomes?

Partly

Is the case presented with sufficient detail to be useful for other practitioners?

Partly

Is sufficient discussion included of the importance of the findings and their relevance to future understanding of disease processes, diagnosis or treatment?

Partly

Is the background of the case’s history and progression described in sufficient detail?

Partly

Reviewer Expertise:

Foot and ankle surgery

I confirm that I have read this submission and believe that I have an appropriate level of expertise to confirm that it is of an acceptable scientific standard, however I have significant reservations, as outlined above.

## References

[1] : Development and reliability of a standard rating system for outcome measurement of foot and ankle disorders I: development of standard rating system. *J Orthop Sci* .2005;10(5) : 10.1007/s00776-005-0936-2 457-65 10.1007/s00776-005-0936-2 16193356 PMC2797841

# Response

**Published:** 2024-04-21

**Authors:** Naoki Kondo

**Affiliations:** Niigata University, Japan

**Points of reviews.**

**Introduction**

 1.In the first paragraph, the authors documented the incidence of postsurgical hallux varus as 2%-15.4%, which does not seem like “very low” to the reviewer. Therefore, the authors are required to reconsider the expression regarding the incidence of iatrogenic hallux varus in the second paragraph.

 →（Response）　I corrected from “very low” to “ not very low”  in the revised manuscript.

**Case report**

 1.JSSF hallux scale consists of three major items, which are pain, function, and alignment. The JSSF scale used in the present study consists of five items, including pain, deformity, ROM, gait, and ADL, which are used for JSSF RA foot and ankle scale (Ref 1). The authors are required to explain the reason why they used JSSF RA scale instead of hallux scale.

 →（Response）　In the present case, we evaluated JSSF RA foot and ankle scale for the functional outcome because the surgery was performed in not only hallux but also lesser toes.

 2.How did the authors evaluate ROM of the hallux? Although the authors mentioned as “the MTP joint was very stiff” and “extension contracture”, the item for ROM was 15 (out of 15 points) preoperatively. The flexion of the hallux was mentioned as -10 in the legend of figure 2, and -30 in the main text.

 →（Response） Thank you so much for the precious comment.

 We reconfirmed the manuscript and legends related to range of motion of MTP joint (preoperatively). Actually, passive extension of MTP joint was 90 °and passive flexion was -30 °. The range of motion was 60°, so this condition was neither “stiff” nor “extension contracture” but “limited range of motion”. So, we corrected the legend of Figure 2 from “-10°“ to “-30°”.

 In addition, the item of ROM was corrected from 15 (out of 15 points) to 13.

 The range of motion of IP joint was also corrected in the manuscript as follows;

 “,but the IP joint remained flexible (-20°in extension and 90°in flexion)(Figures 2A-2D).  

 3.In the 7 ^th^ paragraph, it is also desirable to address the documentation regarding lesser toe shortening osteotomy in more detail.

 →（Response）　Dorsal curved skin incisions were applied in between the 2 ^nd^ and the 3 ^rd^ metatarsals and between the 4 ^th^ and 5 ^th^ metatarsals. Wounds were deepened and extensor digitorum brevis tendons were identified and partially resected. Distal metatarsal regions were subperiosteally released and shortening oblique osteotomy were performed with 45-degrees tilted to the metatarsal longitudinal axis for the 2 ^nd^ through 5 ^th^ toes. The osteotomized thickness were 7 mm. Then, distal fragment was flipped dorsally and osteophyte in the metatarsal head was totally removed and rasped.

 A Kirschner wire of 1.2 mm in diameter was inserted into the metatarsal, and proximal phalanx, mid phalanx, and distal phalanx for each toe.

 Three weeks after the insertion, these wires were removed and weight bearing and gait exercises were performed using arch support. 

**Discussion**

 1.The third paragraph (starting with “Akhtah et al”..) consisting of two sentences may be unnecessary.

 → We deleted the 3 ^rd^ paragraph in Discussion part.

 2.　In the following four paragraphs (4 ^th^ to 7 ^th^), salvage procedures for iatrogenic hallux varus were discussed. However, some of the description referring Leemrijse et al was redundant. Adding the documentation of the indication for MTP preservation as well as MTP arthrodesis will provide readers with a better understanding of treatment algorithm advocated by Leemrijse.

 → （Response）Thank you so much for the precious comment.

 I corrected the part as follows;

 “For procedures of MTP joint preservation, soft tissue release and tendon transfer method using extensor hallucis longus tendon or abductor hallucis longus tendon are used.

 For soft tissue release, medial capsule release or intermetatarsal space release     

 For tendon transfers, 2 methods such as a dynamic transfer and a static transfer are known. The dynamic transfer means transfer with muscle body and the static transfer means transfer with out muscle body. Both transfers compensate for the incompetent lateral collateral ligament. Extensor hallucis longus tendon transfer is selected for a dynamic transfer with or without interphalangeal joint fusion. Abductor hallucis tendon transfer is also used.

 For static tendon transfer, abductor hallucis tendon or artificial implant (TightRope) is selected.　

 When MTP joint is unstable or limited range of motion, MTP joint cannot be preserved , so MTP joint arthrodesis is selected. The present case demonstrated that MTP joint congruity was not so good because of the joint instability and that MTP joint degeneration was clearly detected (Figure 4). Therefore, MTP joint arthrodesis was performed. “.

 3.Was there fibular sesamoid remained? In the paragraph 10, the authors mentioned as “Fibular sesamoid was also released..” On the other hand, fibular sesamoidectomy was supposed to be performed 21 years prior to the revision surgery.

 →　(Response) Thank you so much for the comment. I reconfirmed the operative record. I corrected from fibular sesamoid to tibial sesamoid. The released sesamoid was tibial one.
